# Lieut.-Colonel Simpson Powell

**Published:** 1911-04-01

**Authors:** 


### OBITUARY.

The death is announced at Rangoon, Burma, of Lieut.-Colonel Simpson Powell. He had a distinguished career at Durham University, where he graduated M.D. in 1896, having taken the M.B. with honours in 1883, and his M.R.C.S. a year earlier. He also studied at King's College, of which he was an Associate, before his R.A.M.C. duties took him to India.

